# Examining Factors of Engagement With Digital Interventions for Weight Management: Rapid Review

**DOI:** 10.2196/resprot.6059

**Published:** 2017-10-23

**Authors:** Emma Elizabeth Sharpe, Eleni Karasouli, Caroline Meyer

**Affiliations:** ^1^ College of Life and Natural Sciences University of Derby Derby United Kingdom; ^2^ Division of Clinical Trials Warwick Medical School University of Warwick Coventry United Kingdom; ^3^ Warwick Manufacturing Group University of Warwick Coventry United Kingdom

**Keywords:** weight loss, obesity, patient engagement, self-help devices, health technology, eHealth, mobile apps, patient adherence, review

## Abstract

**Background:**

Digital interventions for weight management provide a unique opportunity to target daily lifestyle choices and eating behaviors over a sustained period of time. However, recent evidence has demonstrated a lack of user engagement with digital health interventions, impacting on the levels of intervention effectiveness. Thus, it is critical to identify the factors that may facilitate user engagement with digital health interventions to encourage behavior change and weight management.

**Objective:**

The aim of this study was to identify and synthesize the available evidence to gain insights about users’ perspectives on factors that affect engagement with digital interventions for weight management.

**Methods:**

A rapid review methodology was adopted. The search strategy was executed in the following databases: Web of Science, PsycINFO, and PubMed. Studies were eligible for inclusion if they investigated users’ engagement with a digital weight management intervention and were published from 2000 onwards. A narrative synthesis of data was performed on all included studies.

**Results:**

A total of 11 studies were included in the review. The studies were qualitative, mixed-methods, or randomized controlled trials. Some of the studies explored features influencing engagement when using a Web-based digital intervention, others specifically explored engagement when accessing a mobile phone app, and some looked at engagement after text message (short message service, SMS) reminders. Factors influencing engagement with digital weight management interventions were found to be both user-related (eg, perceived health benefits) and digital intervention–related (eg, ease of use and the provision of personalized information).

**Conclusions:**

The findings highlight the importance of incorporating user perspectives during the digital intervention development process to encourage engagement. The review contributes to our understanding of what facilitates user engagement and points toward a coproduction approach for developing digital interventions for weight management. Particularly, it highlights the importance of thinking about user-related and digital tool–related factors from the very early stages of the intervention development process.

## Introduction

### Weight Management and Digital Technology

Globally, 39% of the world’s adult population is overweight, and 13% is obese [1]. These rates are increasing, and it is estimated that more than half of the adults will be affected by obesity by 2050 [2]. Obesity is most prevalent in westernized societies such as England, the United States, and Australia. For example, England has one of the highest rates of obesity, with an estimated 62% of the adult population being either overweight or obese [3]. It is well established that obesity is linked to the development of a range of health problems, including type 2 diabetes, coronary heart disease, musculoskeletal disorders, some cancers, and stroke [1,4]. In the United Kingdom alone, this costs the National Health Service in excess of £5 billion per annum [5], and costs arising from the impact of obesity on the wider UK economy (such as loss of productivity) are estimated to be up to £15.8 billion per year [6].

Although a number of interventions for obesity are available (eg, pharmacological treatments and bariatric surgery), these are effective for only a small proportion of the obese population [7]. In addition, these interventions are both costly and associated with a number of adverse physical and psychological effects, including kidney damage [7] and an increase in depression and anxiety [8]. Furthermore, these interventions fail to account for the complexity of eating behavior and the need to promote widespread changes in both diet and physical activity [9]. To initiate and maintain behavior change within overweight and obese populations, interventions must acknowledge the environmental, physiological, and motivational processes that regulate eating and physical activity behaviors [10-13]. However, reported interventions aimed at targeting obesity have had little or no effect on the mounting challenge [14]. Indeed, those who successfully lose weight are likely to regain one-third of the weight lost within the same year and often return to their baseline weight after 3 to 5 years [14,15]. Consequently, there is a clear need for interventions that are able to target daily lifestyle choices (including eating behavior and physical activity) over a sustained period of time.

The need to monitor behavior continuously is crucial for effective behavior change [16]. Therefore, interventions using digital technology may provide one mechanism by which healthy behaviors related to weight management can be targeted throughout a 24-hour period. A digital intervention is a program which aims to offer guidance, information, and support for a variety of physical or mental health programs via a digital platform [17]. Such platforms may take the form of websites, mobile phone apps, or text messages (short message service, SMS). Previously, digital interventions have been successfully developed to help with a number of health-related issues, including the self-management of long-term conditions such as diabetes [18,19], reducing alcohol intake [20], and promoting physical activity [21]. These tools have the potential to provide an attractive method of prompting users to not only change but maintain behaviors with minimal professional contact [22]. As such, digital health interventions are able to provide not only 24-hour availability to self-monitoring statistics, personalized information, and online social support networks but also great affordability, thus, affecting sustained significant change in both a cost- and time-effective manner [23].

Although there has been an increasing interest and investment in digital health interventions [24], their full potential is yet to be realized mainly because of their inability to engage the user into effective and sufficient use [25]. Although user engagement is arguably one of the most important factors in determining the success of an intervention, there are multiple definitions of the construct in the literature. For example, O’Brien et al [26] define user engagement as a quality of users’ experience with technology that is characterized by attributes of challenge, aesthetic and sensory appeal, feedback, novelty, interactivity, perceived control and time, awareness, motivation, interest, and affect. Yardley et al [27], through a process of expert consensus, conceptualized engagement as a dynamic process that usually starts with a trigger (eg, health professional or peers’ recommendations), followed by initial use, and then possibly followed by sustained engagement, disengagement, or shifting to a different intervention. As currently there are no agreed definitions nor validated theoretical models of engagement, for the purposes of this review we adopt a generalized approach that operationalizes engagement as the extent to which people use the digital intervention as intended [28]. The included studies in this review either do not provide information on or they use a variety of engagement definitions. In addition, it is quite often argued that effective engagement should be defined in relation to the purpose of the specific intervention and established empirically in the context of the intervention [27]. Thus, we feel that a more specific definition would be too restrictive for the purposes of this review.

### Aim of the Review

It has been shown that a lack of user engagement with digital health interventions may result in low levels of effectiveness [29-31]. Therefore, improving user engagement with digital tools might result in more effective use and better health outcomes. Thus, it is critical to understand how to better engage potential users with digital health interventions. As different health behaviors are likely to require different engagement strategies [27], this review is focused on weight management and aims to examine users’ perspectives on contributors that are likely to influence engagement with digital interventions and also encourage continued use. Overall, this rapid review aims to synthesize findings from published research to identify possible facilitators and barriers or inhibitors of engagement with digital weight management interventions.

## Methods

### Rapid Review

In recent years, there has been an emergence of rapid reviews within health technology assessments [32]. However, currently, there is no agreed guidance or methodology for rapid reviews. Rapid reviews tend to differ from systematic reviews, in that they are conducted within condensed timelines but follow the main principles of systematic reviews or preferred reporting items for systematic reviews and meta-analyses (PRISMA) guidelines, such as an explicit and reproducible methodology, a systematic search, and a systematic presentation [33]. In the absence of clear guidance, the Cochrane Rapid Reviews Methods Group [34] has been formed to better inform rapid review methodology. Overall, rapid reviews tend to have the following characteristics: they are quicker than systematic reviews (approximately 6-8 weeks); the research question is specified a priori (may include broad PICO [population/patient, intervention/indicator, control/comparator, and outcomes] criteria); sources may be limited but sources or strategies made explicit; exclusion or inclusion criteria are defined either a priori and/or post hoc; they involve rigorous critical appraisal; they may include various depths of syntheses, for example, narrative synthesis and mapping or categorization of the data; and they involve cautious interpretation of the findings to answer the research question. The above methods have been framed based on a number of methodological reviews [35-37]. Given the lack of formal guidelines, this rapid review closely followed the above framework, incorporating when possible some of the established PRISMA guidelines for systematic reviews (eg, reproducible methodology, systematic search, and presentation) while maintaining the timely manner of rapid reviews.

The rationale for conducting a rapid review arises from a need to answer the specified research question rapidly and efficiently. Whereas systematic reviews may provide a comprehensive synthesis of the data, they are often time consuming and costly to produce. Furthermore, as the field of digital health is constantly evolving and because of the speedy technological advances [24], there is a clear need for rapid reviews to draw relatively rapid conclusions about a specific research question. This rapid review also forms part of the formative work for the development of a novel digital health intervention.

### Search Strategy

An electronic literature search was performed using the following databases: Web of Science, PsycINFO, and PubMed. The search was limited to studies published from January 2000 to October 2015. Earlier papers are not deemed relevant to this review because of the rapidity of technological development. Due to limited time and resources available for translation, only articles published in English were included. For each database search, seven key terms (adherence, engagement, motivation, Web-based, mobile, weight, and intervention) were used to create search criteria by combining terms with either the “OR” or “AND” operator (ie, adherence OR engagement OR motivation AND Web-based OR mobile AND weight AND intervention). In addition to electronic searches, manual searches were conducted by screening reference lists of included studies.

### Selection Criteria

Studies were eligible for inclusion if they investigated users’ perspectives on engagement with a digital weight management intervention. Studies with interventions, including digital components alongside nondigital components (eg, associated paper copies of toolkits) were included. Those examining intervention effectiveness but not investigating any aspect of engagement with the intervention were excluded from the review, as were articles where a full text or extractable summary could not be located. We focused on studies that drew their samples from Western societies for two main reasons. Cultural differences can affect individuals’ health beliefs and consequently their health care participation [38,39]. In addition, others have suggested that cultural differences may impact on digital engagement, and so, geographical and cultural differences need to be taken into consideration [40,41]. Finally, as age differences can impact on adoption of technology and user preferences [42,43], any studies using particularly young samples (16 years and under) were excluded.

### Article Screening

One reviewer (ES) screened titles and abstracts using the inclusion and exclusion criteria. When there was uncertainty, a second reviewer (EK) was also consulted. The raters achieved 90% agreement [44]. Disagreements were discussed and resolved by consensus [45]. Full texts of potentially eligible studies were then screened by the first reviewer (ES) and verified by the second reviewer (EK).

### Data Extraction and Synthesis

Data were extracted from relevant publications by one reviewer (ES) using a specially designed data extraction form that was developed according to the Centre for Reviews and Dissemination [45] guidance. The data extraction form collected information on the characteristics of each study, the results as reported by the authors, and key messages (focusing specifically on information relating to facilitators and barriers of engagement with digital weight management interventions). Data from each study were tabulated to compare and aggregate methods, sample characteristics, and research outcomes. Due to the variability in study designs, a narrative synthesis of the data were conducted.

### Quality Assessment

For the purposes of this study, the short electronic health (eHealth)-specific Quality Assessment Checklist was used. The checklist was adapted for the requirements of the rapid review based on the eHealth-specific Quality Assessment Checklist that was originally developed by one of the authors for eHealth-related systematic reviews [46]. The eHealth-specific Quality Assessment Checklist followed the Centre for Reviews and Dissemination [45] guidance, with specific focus on publication-specific contextual, practical, and methodological issues associated with studies describing digital interventions or apps. Studies were assessed according to up to 8 criteria (depending on study design and focus): clear description of purpose, appropriateness of study design, primary methods, digital intervention development process, theoretical frameworks used, users’ description, access, and digital intervention description, including access requirements and intervention components (see Multimedia Appendix 1). No publications were excluded from the review based on quality. This form was independently tested by 2 reviewers (ES and EK) who achieved 91% agreement. Disagreements were discussed and resolved by consensus [45].

## Results

### Study Selection

**Figure 1:**
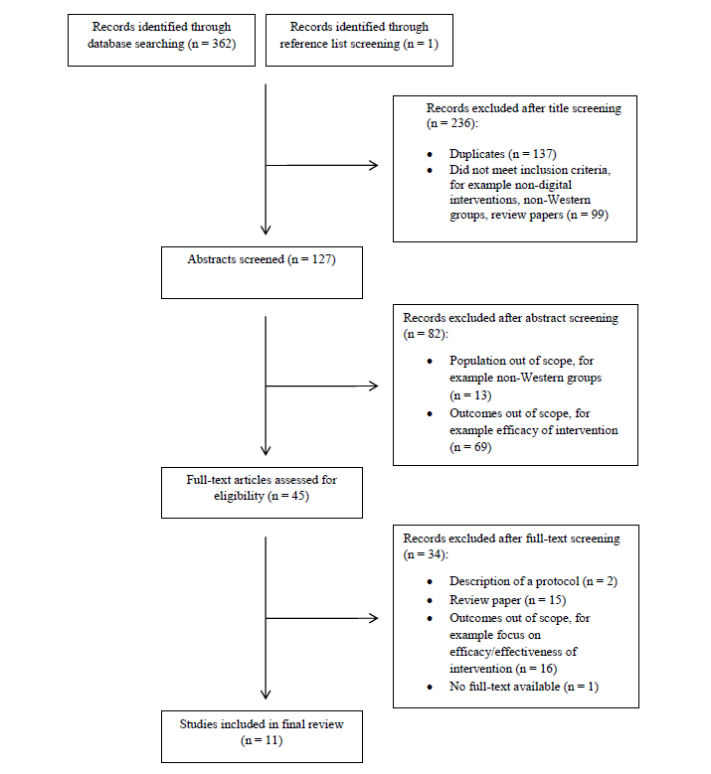
Preferred reporting items for systematic reviews and meta-analyses (PRISMA) flow diagram of study selection process.

Figure 1 shows the results of the screening process. Searching the electronic databases yielded a total of 362 records of which 10 articles met the inclusion criteria. One additional study was identified through reference list screening.

### Quality Assessment

The quality of the papers included in this review varied (see Table 1). Although the majority of included papers investigate users’ engagement with specific digital interventions for weight management, two examine general factors likely to influence engagement with digital weight management interventions [22,47]. Consequently, some of the quality assessment rating items were not applicable to these studies (eg, quality criteria relating to the development process and theoretical underpinning of specific interventions), and thus, they have a maximum quality rating of 4. All other included studies have a maximum quality rating of 8. No studies were excluded based on their quality score or their study design.

**Table 1:** Main characteristics and findings of included studies.

Study	Study design	Quality rating	Digital intervention	Sample	N	Main findings
Brindal et al, 2012 [50]	RCT^a^	7/8	Web-based total well-being diet; targets weight loss. 3 groups: Information-based: dietary and activity-related information provided in a static noninteractive format. Supportive: social interactive website (offers social support in addition to basic information). Personalized-supportive: supportive version with a personalized meal planner.	Adults (18 years or over), BMI^b^>25	8112	Inclusion of social networking features and personalized meal planning did not promote user weight loss or retention but increased average number of user engagement days. In the supportive website, greater use of weight tracker tool led to greater weight loss.
Collins et al, 2013 [51]	RCT	7/8	12-week Web-based weight loss program: The Biggest Loser Club. Basic program: targets self-efficacy, goal setting, self-monitoring, outcome expectations, and social support. Enhanced program: includes all basic features plus personalized features (in response to a behavioral survey), weekly personalized feedback, and an escalating reminder schedule.	Adults (18-60 years), BMI 25 to 40	301	Personalized e-feedback in the enhanced program provided limited additional benefits compared with a standard Web-based weight loss program. However, it supported greater engagement or greater usage, which was related to weight loss.
Dennison et al, 2014 [54]	RCT	8/8	Web-based management intervention: POWeR. Aims to empower users through the development of new self-regulation skills. Coaching calls used to promote continued usage of the POWeR website and adherence to the recommendations within the website.	Adults, BMI>23	786	Usage of POWeR was poor. However, supplementing Web-based weight management with brief human support improved adherence and health outcomes.
Gorton et al, 2011 [47]	Mixed-methods study	4/4	Telephone survey (comprised questions exploring the nature or acceptability of any potential mobile weight loss program). Focus groups explored issues of acceptability.	Individuals over 16 years	306 survey, 54 in focus groups	Participants valued ready access to weight loss information, along with customized feedback and encouragement. Social support, tailored content, and practicality were also identified as features likely to predict engagement.
Lyden et al, 2013 [48]	Qualitative study	6/8	Web-based evidence-based lifestyle intervention.	Adults, BMI>25	50	Participants valued Web-based lifestyle coaching, self-monitoring tools, and structured lesson features. Moderated chat sessions and Web-based resources were rarely used.
McConnon et al, 2009 [55]	Questionnaire-based evaluation of an RCT	8/8	Web-based weight management intervention. The website encourages healthy lifestyle changes, provides information, tools, and support on nutrition and physical activity, as well as behavioral components.	Adults (18-65 years), BMI>30	111	The support sections were used least often and rated most negatively by users. However, poor Internet access may have limited use, thereby reducing the support available to participants.
Mhurchu et al, 2014 [56]	RCT	8/8	12-week weight management program. Comprised of 3 modules (designed to be integrated): Text messaging: Participants sent an average of 2 texts per day over the intervention period. All messages were personalized or tailored toward specific needs (eg, whether they had children). A hard copy toolkit: served as a source of detailed information (able to support personal plans and behavior monitoring). Website: provided a blog to enable participants to share their stories and experiences.	Adults, BMI>25	36	Participants reported that they valued text messages; they found them motivational and liked their clear practical tips and reminders. However, others indicated that they found the messages impersonal, generic, or repetitive.
Morrison et al, 2014 [49]	Mixed-methods study	8/8	POWeR Tracker (weight management app) and POWeR (Web-based weight management intervention). Offers a flexible to foster autonomy and support users to adopt healthy behaviors.	Adults (18-52 years), BMI>23	13	Participants found it convenient to access information on-the-go via their mobiles compared with a computer. However, participants varied in their usage of the Web- versus app-based components.
Patrick et al, 2009 [53]	RCT	7/8	The intervention included personalized short message service and multimedia messaging service messages (sent 2-5 daily) and phone calls (monthly) from a health counselor.	Adults (25-55 years), BMI>25 to 39.9	65	Overall, satisfaction with the intervention was high. Specifically, users found texting their weight every week useful, as it “kept them focused.”
Tang et al, 2015 [22]	Qualitative study	4/4	Semistructured interviews to explore participant experiences of using weight loss apps.	Adults (18-40 years)	19	Participants valued an attractive user interface. Structure, ease of use, personalized features, and accessibility (including dual phone-computer access) were important, and users indicated that continued use depended on these features.
Watson et al, 2015 [52]	RCT	7/8	Imperative health consists of a Web-based program. Designed to assist with lifestyle change (specific focus on diet and nutrition, physical activity, and managing weight).	Adults (over 18 years), BMI 27 to 40	65	Interactivity was essential for engagement. Indeed, the authors argued that the provision of individualized support rather than automated feedback may have helped engagement levels.

^a^RCT: randomized controlled trial.

^b^BMI: body mass index.

Two of the included papers provide a qualitative investigation of specific factors leading to increased engagement [22,48]. Both articles achieved high quality assessment scores, with one achieving 6 out of 8 [44] and the other achieving 4 out of a possible 4 [22]. Two papers explore factors facilitating engagement using a mixed-methods approach [47,49], achieving scores of 4 out 4 [47] and 8 out of 8 [49]. The remaining seven papers are randomized controlled trials (RCTs) and achieved relatively high scores ranging from 7 to 8 out of a possible total score of 8. The average quality rating for studies exploring factors influencing engagement with a specific intervention was 7.33 (standard deviation [SD] 0.71). The mean quality score for studies investigating user engagement with a hypothetical digital weight management intervention was 4 (SD 0). Four out of nine papers lacked information about the intervention developmental process [48,50-52], and one paper lacked information about the theoretical underpinnings used to support the design of their digital intervention [53].

### Characteristics of Included Studies

The characteristics of studies included are summarized in Table 2. Two studies provided a qualitative investigation of the factors that motivate use and encourage engagement with digital interventions for weight management [22,48]. Two studies carried out a mixed-methods investigation of the factors associated with user engagement [47,49]. The seven RCTs examined participant satisfaction and engagement after taking part in a weight management intervention [50-56]. Five of these studies explored engagement with Web-based weight management interventions, three examined participant engagement when using mobile phone weight loss apps, and three examined this when using text message reminders. All included studies were published from 2009 to 2015, with just under half of them (5/11) published on or after 2014 [52-56]. The studies were predominantly carried out in the United Kingdom (5/11) [22,49,52,54,55]. Participants in the included studies were predominately middle-aged, white, and female [47-51,53-56]. Two notable exceptions are the study carried out by Tang et al [22], which aimed to recruit young adults (aged 18-30 years) exclusively and the study conducted by Watson et al [52] in which equal numbers of males and females took part. Most participants in the included studies were in full-time employment [48,49,51,52,54,55]. However, not all the studies provided sufficient information to obtain a detailed educational or employment profile of the sample studied [22,47,50,53].

### Main Findings

The findings are presented according to two key topic areas: factors that influence initial *motivation* to download and/ or use a digital intervention and those that influence subsequent *engagement* with a digital intervention. Table 3 summarizes the main findings.

### Factors That Initially Motivate People to Download and Use Digital Weight Management Interventions

The review identified only one study [22] that explored why people decide to use a weight management digital intervention. Two main areas are highlighted by the study as important motivators.

**Table 2:** Characteristics of included studies (N=11).

Characteristics		n (%)
**Type of study**		
	Randomized controlled trial	7 (64)
	Qualitative study	2 (18)
	Mixed-methods study	2 (18)
**Type of intervention**		
	Web-based	5 (46)
	Mobile phone app	3 (28)
	Text message reminders	3 (28)
**Country**		
	United Kingdom	5 (46)
	Australia	2 (18)
	New Zealand	2 (18)
	United States	2 (18)

**Table 3:** Summary of the main findings.

Initial motivation factors for downloading and using digital weight management interventions	Subsequent engagement factors for enhancing use with digital weight management interventions
Perceptions of one’s physical attractiveness	Personalization
Health outcomes	Social support
	Feedback and encouragement
	Ease of set-up and use
	Self-monitoring and prompts
	Accessibility of information/knowledge

#### Perceptions of One’s Physical Attractiveness

Many users were motivated to use a digital intervention to lose weight to enhance their physical attractiveness, increase their confidence, or generally feel better about themselves [22]. In some cases, motivation to lose weight and download a digital weight loss intervention was prompted by an upcoming social situation or event for which participants wanted to look good or fit into a specific piece of clothing. As expected, this goal-specific incentive to lose weight was linked to sustained digital intervention use and subsequent weight loss.

#### Health Outcomes

Improving health also appeared to be an important initial motivator for wanting to download and use a digital intervention. Specifically, several users identified concerns over health and fitness as the main reason for wanting to download and use a digital weight loss intervention [22]. The motivation to lose weight was particularly apparent if individuals saw themselves as being at an increased risk of health problems (such as diabetes or cardiovascular disorders) because of their weight or family history.

### Factors That Subsequently Enhance User Engagement With Digital Weight Management Interventions

#### Personalization

The findings highlight the importance of *personalization* and the tailoring of interventions to individual needs and goals [47]. *Tailoring* has been described by Kreuter et al [57] as the attempt to reach one specific individual based on particular characteristics of a person that have been assessed or measured beforehand. Specifically, participants expressed a concern over digital interventions, fearing that the tool would be too impersonal. Indeed, when asked to provide follow-up details after completing a digital intervention for weight loss, the characteristics most disliked by participants centered on the generic nature and repetition of the feedback provided [56]. The personal tailoring of information given by digital interventions was also highly valued by users [22]. Interestingly, participants reported that nontailored digital interventions were difficult for them to integrate into their daily routine and expressed frustration at the lack of personal tailoring provided by most digital interventions. Finally, participants were shown to favor the use of personal targets (eg, weight loss, physical activity, and dietary targets) and found them both realistic and motivating [52].

#### Social Support

The importance of social support was emphasized by participants, with many suggesting that digital weight loss interventions should provide a communal aspect to enhance engagement [47]. Indeed, social comparisons may be particularly relevant for the younger adult sample studied [47]. Specifically, links to social networking, a group network, or buddy scheme were all identified as possible ways to incorporate elements of social support into an intervention. These users particularly valued the idea of being able to interact with or contact “someone in the same boat” as them. Furthermore, many participants stated that an awareness of being monitored by others also made them more likely to engage with a particular weight loss intervention [22]. For these users, social comparisons with peers increased their self-efficacy to achieve certain goals.

However, the use of social support within digital weight management interventions has generated mixed responses from participants [47]. For instance, some users reported positive experiences of chat rooms or forums, stating that talking to other users made them feel part of a bigger community, whereas others did not perceive their value. Many users felt chat sessions were not available at convenient times and cited problems with their usability (particularly if the user lacked experience using Web-based chat rooms). Indeed, some users believed that online forums or discussion boards were untrustworthy and felt that they were less likely to use or benefit from that particular feature [22]. The use of chat rooms to foster social support also received the lowest rating score by participants when asked to evaluate their experience of using a digital weight management intervention [55]. However, it is important to note that the use of the site was dependent on traffic to the website, which may have been limited by staggered recruitment into the trial. In this case, the peer support available relied on participants using the website to support each other, and when traffic on the site was low, the social support provided was minimal.

#### Feedback and Encouragement

Regular feedback (on both current behavior and outcomes) and encouragement has been found to be a particularly valued feature [47]. In particular, support from brief telephone coaching can enhance user engagement with digital interventions [54]. Similarly, immediate expert coach feedback has also been shown to be appreciated by many users [48]. In addition, participants were motivated to continue using digital interventions, as they hoped that the feedback provided would lead to effective and sustained weight loss. Specifically, features designed to provide daily encouragement were more likely to facilitate effective behavior change (eg, information regarding calorie intake [too high or low] and successful maintenance of weight goals) [22]. Furthermore, when asked to provide feedback after completing a digital intervention for weight loss, many participants stated that text messages were particularly motivating as they were able to provide clear, practical tips and reminders for changing their eating behavior [56]. However, as previously noted, one of the most common concerns raised regarding daily intervention feedback is that it can be perceived as impersonal, generic, or repetitive [56].

#### Ease of Set-Up and Use

The ability to download and navigate around the digital intervention easily was particularly important to users [22]. When the digital intervention did not seem straightforward to use, the users would describe it as “off-putting” and no longer use it. During follow-up interviews, users also stated that they valued the easiness of initial set-up and suggested that this promoted engagement [52]. Therefore, when digital tools were found to be time-consuming and burdensome, users were less inclined to persevere with the intervention. Specifically, participants noted that tasks that involved uploading and manually entering measurements were particularly tedious and were less likely to encourage adherence in the long term.

#### Self-Monitoring and Prompts

The provision of Web-based self-monitoring tools were particularly well received by users [48,50]. Specifically, daily feedback on eating behavior or weight loss monitoring in the form of graphs or pie charts was perceived as particularly helpful. Self-monitoring was also valued by participants who rated this particular feature above all other components [55]. When asked to provide follow-up comments after completing a digital intervention, participants also acknowledged the importance of self-monitoring and found the use of reminders and daily weight texts especially beneficial [53]. Finally, users emphasized the usefulness of frequent notifications of reminders in facilitating effective behavior change [22]. These were reported to be particularly effective in motivating health behavior when sent as a personalized message to prompt action.

#### Accessibility of Information or Knowledge

Participants emphasized the need for a digital weight loss intervention to include relevant content with both practical and achievable messages [47]. They also highlighted the need for digital interventions to address the psychological aspects of weight loss, including the emotional and external factors associated with overeating. One of the main advantages participants described of using digital weight loss interventions was the fact that relevant information was “at your fingertips,” and they particularly valued the flexible means by which this information could be accessed. Users also saw information about lifestyle change and behavior modification as a particularly useful feature within digital weight loss interventions [48]. Indeed, the provision of accessible links to reliable Web-based resources was one of the most highly rated features of the particular intervention.

## Discussion

### Summary of Results

This rapid review aimed to provide a brief synthesis of the factors that influence user engagement with digital weight management interventions. The papers included varied in design, with two providing a qualitative investigation, two utilizing a mixed-methods approach, and seven conducting an RCT. The findings revealed a distinction between the initial motivation to download and first use a digital intervention and the subsequent engagement with the digital intervention over time. According to our findings, different factors seem to influence the two phases of motivation and engagement. The first phase (motivation) where a potential user decides whether to use a digital intervention for the first time is influenced by user related characteristics (eg, self-perceptions of body and health outcomes). The second phase (engagement) where the user continues using, some or all, of the elements of the digital intervention is influenced predominately by characteristics related to the digital intervention (eg, ease of use and the provision of personalized information). Overall, the quality of the studies included in this review was high.

### Factors Facilitating Initial Motivation

The findings emphasized the importance of user-related factors in an individual’s initial motivation to download or visit a digital intervention with the aim of using it for weight management. In a Delphi experiment, Brouwer and colleagues [58] also found that user characteristics are important in this first phase of using a digital intervention. In the same way, recent research has demonstrated that an intrinsic motivation to better their health may encourage users to persist with a digital intervention [59]. Specifically, in this review, it was found that individuals were motivated to use a digital intervention to lose weight, improve their health, and/or enhance their perceived physical attractiveness. Expectedly, those who were motivated to lose weight were often the ones who were most successful in maintaining their weight loss [22]. As a result, it may be useful to incorporate motivational enhancement techniques to encourage use of weight interventions. Gauging participant views and attitudes beforehand may also help to predict those who are more likely to use the intervention. On the basis of the fact that the review identified only one study looking at motivation around using digital weight management interventions and as, according to the authors’ knowledge, there are no related studies for other health behaviors, there is a clear need of further research to improve our understanding around motivation and its potential relationship with engagement.

### Factors Facilitating Subsequent Engagement

In agreement with Brouwer et al [58], this review shows the importance of specific digital intervention features in facilitating an individual’s engagement with a digital intervention. More specifically, the personalization of the digital intervention, such as providing individualized feedback and encouragement, was linked to higher levels of engagement across a range of studies [22,47,52,54,56]. This is consistent not only with more recent findings [59] but also with an earlier review suggesting that tailored advice and feedback improves user engagement with digital health interventions [60]. In addition, previous research emphasizes the association between the provision of personalized information and weight loss [61,62]. Therefore, providing participants with information specific to their individual circumstances and needs over and above the provision of generic, repetitive feedback may play a crucial role in facilitating effective behavior change. This may be achieved by obtaining detailed user information and gauging specific health and weight goals during set-up.

Access to social support (eg, peer groups) through the digital intervention was also highly valued by users across a range of studies [22,47,48]. Specifically, the availability of social support at any time and location was shown to promote engagement by making users feel valued and supported throughout the intervention [22]. The association between social support and user engagement in digital health interventions has been established in both the review by Brouwer [63] and Schubart’s study [60] but not in Kelders et al’s [28] review. However, it should be noted that in the review carried out by Kelders et al [28], social support referred to those interventions providing only the *opportunity* to contact others and did not measure how frequently this particular feature was used. In other words, where an intervention included a discussion board or forum, this was categorized as social facilitation, even when no posts were made. As such, not all offers of social support may have been taken up by users, which may help to explain the lack of positive association in this case. In addition, the link between social support and successful weight management is well established [64-66]. Thus, future digital interventions could benefit from incorporating a social support element into their design. Nevertheless, findings from this review revealed that social support relies heavily on other users sharing similar views and experiences, and when the support provided from other users is minimal, its role in encouraging engagement is decreased [55]. It might be the case that the type of social support valued may differ depending on specific user characteristics. Therefore, designers may choose to make such features available but optional within future weight management interventions.

Self-monitoring features within digital weight management interventions were also associated with enhanced engagement [48,50,55]. Previously, research has demonstrated that eating behavior monitoring is necessary for effective behavior change [67,68]. However, constant self-monitoring can be monotonous and difficult to sustain. Using digital technology to prompt an individual to monitor or engage in a particular behavior may therefore provide an advantage over interventions that do not offer such features [22]. The importance of such features in encouraging continued use of a digital intervention [22,50,53] also highlights the key role of habituation in maintaining successful behavior change. In this way, daily prompts or reminders may increase the likelihood that a certain behavior becomes habitualized and incorporated into an individual user’s daily routine [69]. This has also been supported by more recent research which highlights the role of prompts (specifically email reminders) in promoting continued user engagement with digital health interventions [70].

Finally, ease of using the digital interventions was found to be an important facilitator of enhanced engagement [22,52]. Notably, participants reported that they found non-user-friendly and nontailored digital interventions difficult to integrate into their daily routines [22]. Aesthetically attractive digital interventions that were easy to set up and use were among those rated most highly by users. Again, this closely aligns with recent work demonstrating a relationship between user-friendly technology and increased engagement with digital interventions for health [59]. Participants also found those that providing personal tailoring (ie, the opportunity to customize the digital intervention by changing colors and images, etc) to be more satisfying to use, and as a result, they were more likely to engage with these interventions for longer. This supports previous findings, which have emphasized the importance of personal tailoring in facilitating continued engagement with digital interventions [71]. Taken together, these findings suggest that future digital intervention designs should focus on key areas such as product functionality, user autonomy, and personalization.

### Strengths and Limitations of the Review

To our knowledge, this is the first review to examine factors that may facilitate motivation to use and also further engagement with digital weight management interventions. The findings are summarized taking a user perspective and looking at the user’s experiences and perceptions, thus providing useful recommendations for behavior change researchers and digital intervention developers. A key strength of this rapid review is the aim to minimize the risk of bias through the use of quality checklists and criteria (in line with PRISMA guidelines). Nevertheless, there are a few notable limitations. The review focuses predominately on samples living in Western societies, as cultural differences may influence health beliefs and engagement with one’s health care and digital interventions [38-43]. Thus, even though this specific focus gives a clear picture for Western societies, the findings may be interpreted with caution to non-Western societies. The electronic literature search performed identified studies published until October 2015, so any studies published at a later date are not necessarily captured in the results of this review. However, we later performed an updated search in PsycINFO for any studies published until September 2017, and no additional studies were identified. Furthermore, as rapid reviewing is a relatively new methodology, currently, there are no agreed guidelines that may be followed. There is also a possibility that relevant literature may have been missed because of the rapidity of the search process, so a natural step following the findings of this review would be to conduct a comprehensive, systematic literature review. A framework based on relevant methodological reviews [35,37,72] is, however, available, and this rapid review closely followed this. Given the limited number of studies published in this area and the constant technological advancements, the rapid review method was deemed most appropriate. Indeed, the fast-paced nature of this review addresses a clear need to draw rapid conclusions about specific research questions within the constantly advancing field of digital health. In addition, it is worth noting that Watt et al [73] found that despite “axiomatic differences” between systematic and rapid reviews, “the essential conclusions of the rapid and full reviews did not differ extensively.”

### Research and Practical Implications

The findings of this rapid review clearly highlight the need for further research to better understand what motivates people in using digital weight management interventions and what makes them engage with such interventions over a sustained period of time. Such understanding will potentially come from focusing on specific groups of people because of the variety of unique characteristics and needs. For example, age—although not measured in the present review—might be a possible contributing factor warranting further research. Younger people tend to be more familiar with using technology in their everyday lives, and therefore, their needs, perceptions, and mastery levels may differ to those of older individuals who may not necessarily have integrated new technologies in their lives [39]. For this, exploratory studies using qualitative methods could be ideal in furthering our understanding in both motivational and engagement issues but also in the relationship between the two.

As currently, most digital weight management interventions fail to sustain user engagement and subsequently to achieve and maintain positive health behavior change, focus should be placed not only on effective behavior change techniques that are relevant to the health behavior of interest but also on how to enhance engagement with the intervention. The findings from this review should therefore be taken into consideration by incorporating specific features or components most valued by users when developing future digital weight management interventions. The development process could particularly benefit from an element of coproduction with key stakeholders and users and also of allowing for intervention personalization or tailoring.

### Conclusions

Digital weight management interventions provide a unique opportunity to offer tailored help, support, and guidance for weight management; however, currently, the full potential of such interventions is hindered by a lack of user engagement. This review helps to further our understanding of the key issues around user engagement and points toward a coproduction approach for developing digital health interventions. Particularly, it highlights the importance of considering both user-related and digital tool–related factors from the early stages of the development process.

## Appendix

Multimedia Appendix 1 Quality assessment checklist.

## Abbreviations

eHealth: electronic health
BMI: body mass index
PICO: population/ patient, intervention/ indicator, comparator/ control, outcome
PRISMA: preferred reporting items for systematic reviews and meta-analyses
RCT: randomized controlled trial
SD: standard deviation
