# Supplementary material for: Examining Factors of Engagement With Digital Interventions for Weight Management: Rapid Review
Source: JMIR Res Protoc. 2017 Oct 23;6(10):e205. doi: 10.2196/resprot.6059 (PMC5673884; doi:10.2196/resprot.6059)
Supplement: Multimedia Appendix 1 [file resprot_v6i10e205_app1.pdf]

Quality Assessment Checklist – Systematic Review – digital interventions/apps

Quality Criteria											
	**Brindal (2012)**	**Collins (2013)**	**Dennison (2014)**	**Gorton (2011)**	**Lyden (2013)**	**McConnon (2009)**	**Mhurchu (2014)**	**Morrison (2014)**	**Patrick (2009)**	**Tang (2015)**	**Watson (2015)**
**Purpose (clear description of the purpose incl aims of study/digital intervention/app)**	Yes	Yes	Yes	Yes (basic)	Yes	Yes	Yes	Yes	Yes	Yes	Yes
**Study design (appropriateness according to development & evaluation process for mhealth i.e. focus group, pre-testing, pilot, RCT)**	Yes (RCT)	Yes (RCT)	Yes (RCT)	Yes (mixed method- focus groups & telephone survey)	Yes (qualitative study)	Yes (RCT)	Yes (RCT)	Yes (mixed methods)	Yes (RCT)	Yes (qualitative study)	Yes (RCT)
**Main methods (clear description of study's methods and analysis)**	Yes (participants randomly allocated to groups)	Yes (participants randomly allocated to groups)	Yes (participants randomly allocated to groups)	Yes	Yes (semi-structured interviews)	Yes (participants randomly allocated to groups)	Yes (participants randomly allocated to groups)	Yes (telephone interviews & usage)	Yes (participants randomly allocated to groups)	Yes (semi-structured interviews)	Yes (participants randomly allocated to groups)

**History/ development process of digital intervention/app (clear description incl formative evaluations, i.e. focus groups, usability testing)**	No	No	Yes (link provided to earlier paper)	N/A	No	Yes	Yes	Yes	Yes (info on formative research/focus groups used to inform the design the intervention)	N/A	No
**Theory/evidence base used to design the digital intervention/app (clear description of theoretical framework used)**	Yes (behaviour change: health action process approach)	Yes (social cognitive theory)	Yes (various theoretical models/behaviour change techniques: not specified)	N/A	No	Yes	Yes	Yes	No	N/A	Yes
**Users (description of participants/aimed users incl target population, age range)**	Yes	Yes	Yes	Yes (participant information provided)	Yes	Yes	Yes	Yes	Yes	Yes	Yes
**Access (description of how participants accesses the digital intervention/app, in what setting/context, if they had to pay)**	Yes	Yes	Yes	N/A	Yes	Yes	Yes	Yes	Yes	N/A	Yes

**Features (description of digital intervention/app features & components)**	Yes	Yes	Yes	N/A	Yes	Yes	Yes	Yes	Yes	N/A	Yes
**Total Score**	7/8	7/8	8/8	4/4	6/8	8/8	8/8	8/8	7/8	4/4	7/8
